# Lineage tracing reveals the hierarchical relationship between neural stem cell populations in the mouse forebrain

**DOI:** 10.1038/s41598-019-54143-9

**Published:** 2019-11-27

**Authors:** Nadia Sachewsky, Wenjun Xu, Tobias Fuehrmann, Derek van der Kooy, Cindi M. Morshead

**Affiliations:** 1 Department of Surgery, Division of Anatomy, University of Toronto, Ontario, Canada; 2 Institute of Medical Sciences, University of Toronto, Ontario, Canada; 3 Department of Molecular Genetics, University of Toronto, Ontario, Canada; 4 Institute of Biomaterials and Biomedical Engineering, University of Toronto, Ontario, Canada; 5 Toronto Rehabilitation Institute, University Health Network, Toronto, Ontario Canada

**Keywords:** Developmental neurogenesis, Neural stem cells

## Abstract

Since the original isolation of neural stem cells (NSCs) in the adult mammalian brain, further work has revealed a heterogeneity in the NSC pool. Our previous work characterized a distinct, Oct4 expressing, NSC population in the periventricular region, through development and into adulthood. We hypothesized that this population is upstream in lineage to the more abundant, well documented, GFAP expressing NSC. Herein, we show that Oct4 expressing NSCs give rise to neurons, astrocytes and oligodendrocytes throughout the developing brain. Further, transgenic inducible mouse models demonstrate that the rare Oct4 expressing NSCs undergo asymmetric divisions to give rise to GFAP expressing NSCs in naïve and injured brains. This lineage relationship between distinct NSC pools contributes significantly to an understanding of neural development, the NSC lineage *in vivo* and has implications for neural repair.

## Introduction

The adult mammalian central nervous system contains two distinct populations of neural stem cells (NSCs). Within the well-documented neurogenic niche of the adult forebrain germinal zone, glial fibrillary acidic protein (GFAP) expressing definitive NSCs (dNSC) infrequently divide with a cell cycle time of 2–4 weeks to produce more rapidly dividing progenitors that migrate along the rostral migratory stream towards the olfactory bulb and generate interneurons^1–4^. A second population of primitive NSCs (pNSCs), initially isolated during development as early as embryonic day (E) 5.5, persists into adulthood. The pNSC does not express GFAP, but does express low levels of the pluripotency marker, Oct4^5–7^. Primitive NSCs are rare (more than 10 fold less frequent than dNSCs) and have a long cell cycle time of 3–5 months in the adult brain^4,7^.

*In vitro*, dNSCs proliferate in the presence of epidermal growth factor (EGF) and fibroblast growth factor (FGF) to form clonally derived colonies of cells termed neurospheres^8^. pNSCs generate clonally derived, multipotent colonies in the presence of leukemia inhibitory factor (LIF)^6,7^. Definitive and primitive NSCs can be further differentiated based on the expression of cell surface markers c-kit and ErbB2^9^. Previous work supports the hypothesis that pNSCs are lineally related to dNSCs^7^. First, *in vitro* culture experiments demonstrate that adult-derived pNSC colonies can be induced to form dNSC neurospheres (in EGF + FGF + heparin, EFH) but not vice versa, and second, transplantation of pNSCs onto the rostral migratory stream (RMS) revealed pNSC derived neuroblasts on the RMS and neurons in the olfactory bulb. Further, Reeve *et al*.^4^ showed that in conditional Oct4 loss of function mice (Oct4fl/fl;Sox1Cre), repopulation of the dNSC population following ablation does not occur. These findings support the hierarchical lineage relationship between pNSCs and dNSCs.

Herein we have used several transgenic mouse models that permit lineage tracking of pNSC progeny in embryonic, neonatal and adult mice. Using labeling paradigms in the developing CNS, in combination with *in vivo* ablation paradigms in the adult, we demonstrate that pNSCs are the precursors to dNSCs *in vivo*. Retroviral lineage tracing and a triple transgenic mouse (Oct4CreER^T2^;ROSAyfp^fl/fl^;GFAPtk) that permits pre-labeling of the very rare Oct4+ cells *in vivo*. We show that Oct4+ pNSCs can give rise to dNSCs in the adult mouse periventricular region in naïve brains and in a loss of function model of dNSC repopulation.

## Results

### Embryonic pNSCs give rise to dNSCs and all neural cell types in the brain

**Figure 1:**
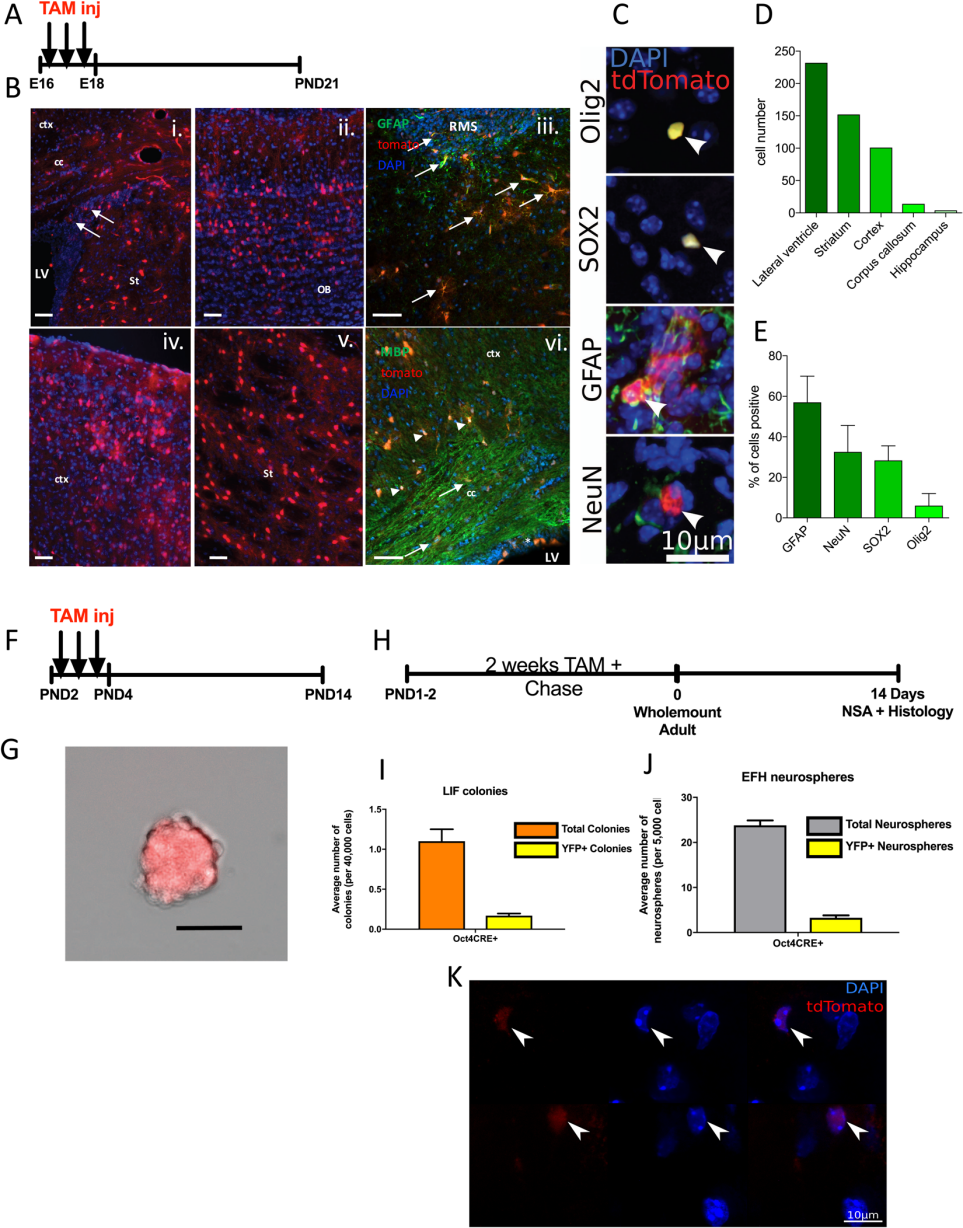
Identification of pNSCs in the developing and adult forebrain. (**a**) Schematic of the experimental paradigm for labeling Oct4 + pNSCs during embryogenesis (E16-18). (**b**) Sections showing tdTomato+ cells (red) throughout the brain of PND21 mice in (i) the germinal zone adjacent to the lateral ventricles (LV), (ii) olfactory bulb (OB), (iii) rostral migratory stream (RMS) with adjacent cells co-localized with GFAP (green; arrows), (iv) the cortex (ctx), (v) the striatum (St), and (vi) the corpus callosum (cc, arrow) and cortex (ctx, arrowhead) co-localized with the marker for MBP (green). Scale bars = 50 µm. blue = Hoescht. (**c**) High magnification, confocal images of all lineages identified at PND21 that co-localize with tdTomato+, pNSC derived cells including oligodendrocytes (Olig2), undifferentiated progenitors (Sox2), astrocytes (GFAP), and neurons (NeuN). (**d**) Regionalization of tdTomato+ cells throughout the brain. (**e**) Relative percentage of various cell types within the tdTomato+ cells located in the PND21 brains. (**f**) Schematic of experimental paradigm for labeling Oct4 + pNSCs during early post-natal period (PND2-4). (**g**) Representative image of a TdTomato+ LIF colony at PND14 following early post-natal labeling. Scale bar = 50 µM. (**h**) Schematic of experimental paradigm for labeling pNSCs postnatally. (**i**) At 4 weeks post-TAM the numbers of pNSCs (LIF) (orange bars) and co-expressing YFP+ neurospheres is indicated in yellow bars (n = 5 mice/group). (**j**), At 4 weeks post-TAM the total numbers of dNSCs (grey bars) and co-expressing YFP+ neurospheres (yellow bars) was assessed (n = 5 mice/group). (**k**) Wholemount of the lateral ventricles at 2 weeks post-TAM induction. Arrowheads indicate Tdtomato+ cells. Blue = DAPI, Red = Tdtomato. All data represents mean ± SEM.

Primitive NSCs express the pluripotency marker Oct4, which distinguishes this population from dNSCs^7^. To determine whether pNSCs give rise to dNSCs *in vivo*, we took advantage of an inducible Oct4CreER^T2^ crossed to a reporter mouse (ROSAyfp^fl/fl^ or ROSA26-tdtomato^fl/fl^) to enable the selective labeling of pNSCs. Using the transgenic reporter line Oct4CreER^T2^; ROSA26-tdtomato^fl/fl^, we labeled the pNSCs when they are most abundant in the developing brain^7^. Timed pregnant females were injected with tamoxifen once a day at gestational age E16–18, a period when the developing brain is expanding the size of the neural precursor pool and undergoing neurogenesis and gliogenesis^10–12^ (Fig. 1a). We predicted that pNSCs labeled in the Oct4CreER^T2^; ROSA26-tdtomato^fl/fl^ mice would generate progeny of all three lineages as well as give rise to dNSCs.

Consistent with the prediction that the pNSC lies at the top of the neural cell lineage, we found tdTomato+ cells, derived from an Oct4 + pNSC, in all areas of the brain at PND21 (Fig. 1b). tdTomato+ cells were found in the germinal zone of the lateral ventricle (Fig. 1bi) where neural precursor cells are found, as well as in the olfactory bulb (Fig. 1bii), the cortex (Fig. 1biv), and striatum (Fig. 1bv). The tdTomato+ cells had the morphology of neurons and subpopulations of cells were co-labeled with glial markers (GFAP and MBP; astrocytes and oligodendrocytes, respectively) (Fig. 1biii,vi). To confirm the derivation of other lineages from pNSC derived cells, high magnification, confocal images confirmed the co-expression of tdTomato+, Oct4 expressing pNSC derived cells of mature phenotypes including oligodendrocytes (Olig2), neurons (NeuN), and astrocytes (GFAP) as well as uncommitted progenitors (Sox2)(Fig. 1c). We found tdTomato+ cells in all Oct4CreER^T2^; ROSA26-tdtomato^fl/fl^ that received TAM (4/4). The tfTomato+ cells differentiated into all lineages examined (Fig. 1d). Interestingly, the bias for the distribution of tdTomato+ cells is towards the germinal zone at this time point, consistent with the idea that these cells are derived from pNSCs (Fig. 1e). Hence, during embryogenesis, rare, Oct4+ pNSCs contribute to neural cells throughout the developing brain.

A separate cohort of Oct4CreER^T2^; ROSA26-tdtomato^fl/fl^ mice received TAM through the mother’s milk to label pNSCs in the early postnatal brain. The mothers were injected with TAM on PND2-4 (Fig. 1f), and the mice were sacrificed at PND14 and processed for the pNSC-derived, tdTomato+ LIF colonies; a time when we have observed significantly more LIF colonies compared to adult mice. Consistent with our predictions, we observed tdTomato+ LIF colonies from all mice examined (n = 8) (4.625 ± 0.73 tdTomato+ LIF colonies/mouse) (Fig. 1g).

Based on these findings in the early postnatal brain, we predicted that labeling pNSCs in Oct4CreER^T2^;ROSAyfp^fl/fl^ mice would result in a cohort of YFP + dNSCs which could be identified in the neurosphere assay in adult mice. Postnatal day 1–2 (PND1–2) mice were exposed to TAM through the mother’s milk for 14 days to maximize the labeling of a cohort of Oct4+ pNSCs, and the neurosphere assay was performed in young adult mice (Fig. 1h). As predicted, YFP+ pNSC-derived colonies were isolated from adult mice (mean = 6.46% of all LIF responsive colonies) (Fig. 1i). We also observed a cohort of YFP+ dNSC-derived neurospheres (mean = 13.87%), indicating that dNSCs were derived from Oct4+ cells labeled in the early postnatal period (Fig. 1j). Most important, no YFP+ neurospheres were derived from cultures of TAM fed Oct4CreER^T2−/−^;ROSAyfp^fl/fl^ littermate controls and non-fed Oct4CreER^T2^;ROSAyfp^fl/fl^ indicating promoter-specific labeling. A separate cohort of TAM fed Oct4CreER^T2^; ROSA26-tdtomato^fl/fl^ mice were perfused and wholemount preparations the lateral ventricles revealed the presence of Tdtomato+ cells in young adult mice (Fig. 1k). Hence, Oct4+ pNSCs can give rise to dNSC progeny *in vivo*, under non-injury conditions.

Taken together, these data demonstrate the lineage relationship between pNSCs and dNSCs under baseline conditions, consistent with work done previously^7^. We next asked whether this lineage relationship persisted into the adult brain when the pNSCs primarily are quiescent *in vivo* but are activated in response to injury^4,7^.

### dNSCs are repopulated by GFAP negative cells

**Figure 2:**
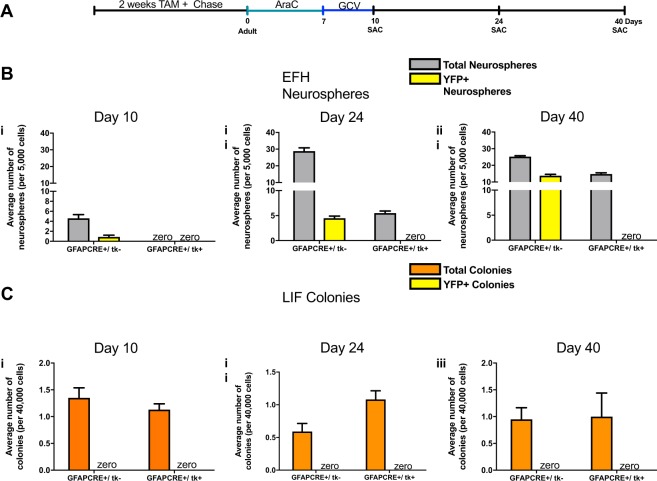
Repopulation of dNSCs from a non-GFAP expressing cell. (**a**) Schematic of the experimental paradigm. (**b**) The neurosphere assay for dNSCs (EFH) (grey bars) performed in control (GFAPCRE+/tk−) and experimental groups (GFAPCRE+/tk+) at day 10 (i), day 24 (ii), or day 40 (iii) after the onset of ablation. The numbers of YFP+ neurospheres are indicated in yellow bars (n = 6 mice/group/survival time). (**c**) The colony-forming assay for pNSCs (LIF) (orange bars) performed in control (GFAPCRE+/tk−) and experimental groups (GFAPCRE+/tk+) at day 10 (i), day 24 (ii), or day 40 (iii) after onset of ablation. The numbers of YFP+ colonies are indicated in yellow bars (n = 6 mice/group/survival time). All data represent mean ± SEM.

Previous studies have shown that the administration of ganciclovir (GCV), with or without the mitotic inhibitor AraC, to GFAPtk mice results in a complete loss of dNSC-derived neurospheres within a few days of treatment^3,7^. However, the AraC and GCV ablation paradigm does not result in a permanent depletion of dNSCs and over time, dNSC repopulation occurs^7^. We postulated that the GFAP negative (pNSC), or a quiescent dNSC (GFAP+), contributed to this repopulation. To address this question we administered tamoxifen (TAM) for 2 weeks to young adult triple transgenic GFAPCreER^T2^;ROSAyfp^fl/fl^;GFAPtk mice (herein termed GFAPCRE+/tk) to label a cohort of GFAP + dNSCs (Fig. 2a). This labeling paradigm resulted in 32–56% of the dNSCs derived clonal neurospheres expressing YFP in both the experimental strain (GFAPCRE+/tk+) and the littermate control strain (GFAPCRE+/tk−).

After establishing baseline labeling, dNSC ablation was performed using AraC and GCV. Mice received 7 days of intraventricular AraC infusion followed by 3 days of intraventricular GCV to selectively and completely ablate dividing GFAP+ cells, as previously described^7^ (Fig. 2a). Immediately following ablation (day 10), there was a complete loss of dNSC-derived, EFH neurospheres from GFAPCRE+/tk+ mice (Fig. 2bi). Control mice (GFAPCRE+/tk−) had a YFP+ cohort (28 ± 11% of the total clonal neurospheres formed) that was not killed by the GCV due to the lack of the tk transgene (Fig. 2bi). As predicted, none of the pNSC, LIF responsive clonal colonies expressed YFP confirming that pNSCs are GFAP negative (Fig. 2ci). At 14- and 30-days post ablation (day 24 and day 40, respectively), the dNSC pool expanded and repopulated the subependyma, as indicated by the increase in total EFH clonal neurosphere numbers. Notably, none of the GFAPCRE+/tk+ dNSC-derived neurospheres expressed YFP, revealing that the dNSC-derived neurospheres did not originate from the previously labeled GFAP+ dNSC cohort (Fig. 2bii–iii). The control mice (GFAPCRE+/tk−) generated EFH neurospheres, and a subset were YFP+ (Day 24 = 16 ± 2%, Day 40 = 55 ± 2% of all of the neurospheres formed) (Fig. 2bii,iii). Most importantly, we never observed YFP+, LIF responsive colonies in AraC+ GCV treated experimental or control mice, at any time examined, confirming their lack of GFAP expression (Fig. 2ci–iii). The number of pNSC derived clonal colonies was not significantly different between groups (Fig. 2ci–iii; two-way ANOVA, p > 0.05).

Furthermore, taking advantage of a GFAP reporter mouse, we performed a related but distinct ablation to examine the precision of ablating the dNSC population. GFAP− gfp mice received intraventricular infusion of 2% AraC for 7 days and immediately sacrificed (Suppl. Fig. 1a). Neurosphere formation from dNSCs in vehicle infused mice were normal; however, in AraC ablated mice, there were no EFH neurospheres, demonstrating an effective elimination of dNSCs (Suppl. Fig. 1b). Not surprisingly, the more quiescent pNSC population was unaffected by the ablation, and LIF colony formation remained at control levels and never expressed GFAP at discernible levels (Suppl. Fig. 1c). *In vivo* sections of these brains reveal that following AraC ablation, there is a loss of GFAP expressing cells in the subependyma of GFAP-gfp mice (Supp. Fig. 1d) not seen in vehicle infused controls (Suppl. Fig. 1e), further confirming the ablation of the dNSC population and their progeny in this model. Taken together, these findings demonstrate that repopulation of the dNSC pool following ablation occurs from a GFAP negative cell.

### Ablation of dNSCs leads to the proliferation of a cohort of pNSCs that contribute to dNSC repopulation

**Figure 3:**
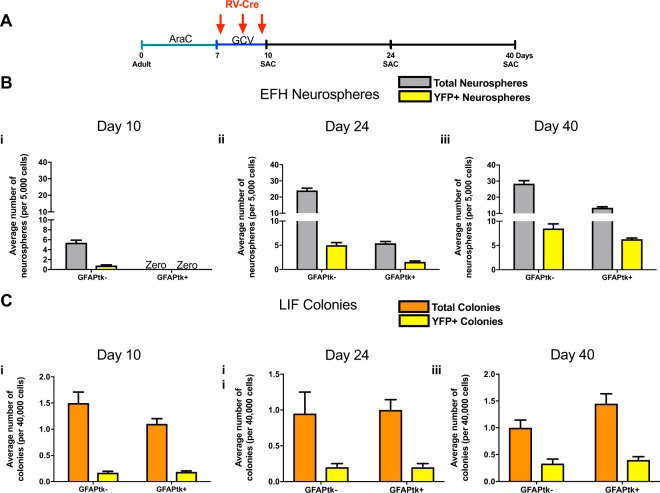
Proliferating, GFAP negative cells give rise to dNSCs following ablation. (**a**) Schematic of the experimental paradigm. (**b**) The neurosphere assay for dNSCs (EFH) (grey bars) performed in control (GFAPtk-) and experimental groups (GFAPtk+) at day 10 (i), day 24 (ii), or day 40 (iii) after onset of ablation. The numbers of YFP+ neurospheres are indicated in yellow bars (n = 6 mice/group/survival time). (**c**) Colony-forming assay for pNSCs (LIF) (orange bars) performed in control (GFAPtk−) and experimental groups (GFAPtk+) at day 10 (i), day 24 (ii), or day 40 (iii) after the onset of ablation. The numbers of YFP+ colonies are indicated in yellow (n = 6 mice/group for day 10; n = 3 mice/group for day 24; n = 4–6 mice/group for day 40). All data represent mean ± SEM.

To demonstrate that a subset of pNSCs are recruited to contribute to the repopulation of the dNSC pool following ablation, a series of experiments were carried out that relied on the proliferative status of the recruited cells during repopulation. We performed retroviral labeling in adult ROSAyfp^fl/fl^;GFAPtk mice that underwent the AraC+ GCV ablation. We injected a Cre-Recombinase expressing retrovirus (RV-Cre) intraventricularly to label proliferating cells during the repopulation phase (Fig. 3a). We predicted that the GFAP negative, pNSCs cells would be recruited and actively proliferate during the repopulation phase, and thus would be labeled with the RV-Cre and express YFP+.

Immediately following the AraC+ GCV ablation (day 10), the GFAPtk+ mice had a complete loss of dNSC-derived neurospheres (Fig. 3bi), as previously shown (Fig. 2). As predicted, control (GFAPtk-) mice had a cohort of dNSCs that were YFP+ (16.93 ± 1.72%), indicating that dNSCs were proliferating post-AraC infusion and were labeled with the RV-Cre (Fig. 3bi). Most interesting, YFP+ pNSC-derived LIF colonies were observed immediately following ablation (day 10) in both GFAPtk+ and GFAPtk- mice, indicating that pNSCs were actively proliferating and infected with RV-Cre during the GCV infusion (Fig. 3ci). At longer survival times post-ablation (days 14 and 30), the dNSC-derived, EFH responsive neurospheres returned in both groups and most importantly, a subpopulation of dNSC-derived neurospheres in GFAPtk+ mice were YFP+ (Fig. 3bii–iii). Thus, the repopulating cell in the experimental group is a GFAP−, proliferating pNSC cell, labeled by the RV-Cre during the GCV infusion (Fig. 3bi–iii).

The GFAPtk- control mice also underwent rapid repopulation of dNSC derived neurospheres, and a subpopulation of the dNSCs are YFP+ (Day 24 = 20.71 ± 1.73%, Day 40 = 29.77 ± 2.39% of all neurospheres). Notably, absolute numbers and the percentage of YFP+ pNSC derived, LIF colonies remained constant at all times examined and in both experimental and control groups (Fig. 3ci–iii) suggesting that pNSCs undergo asymmetric divisions *in vivo*. Together, these findings indicate that pNSCs proliferate in response to dNSC depletion and may serve as precursors for dNSCs.

### Oct4 + pNSCs are responsible for the repopulation of dNSCs

**Figure 4:**
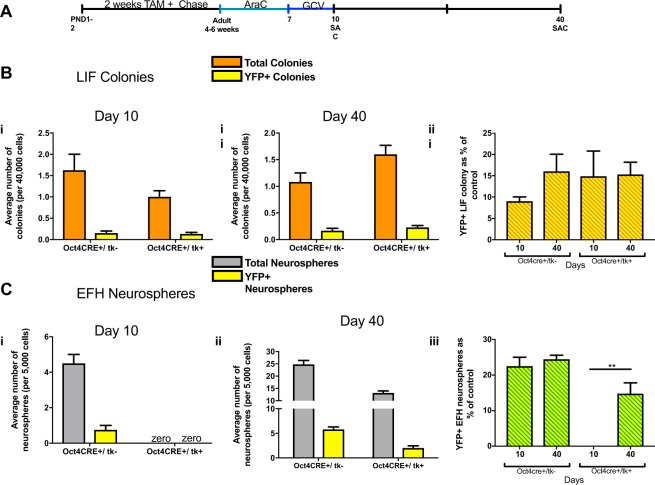
Oct 4 expressing, primitive NSCs give rise to dNSCs following ablation. (**a**) Schematic of the experimental paradigm. (**b**) The colony-forming assay for pNSCs (LIF) (orange bars) was performed in control (Oct4CRE+/tk−) and experimental mice (Oct4CRE+/tk+) at day 10 (i) or day 40 (ii). The numbers of YFP+ colonies are indicated in yellow bars. Day 10, n = 3 Oct4CRE/tk- mice, n = 5 Oct4CRE/tk+ mice; Day 40, n = 5 mice/group. (**c**) The numbers of dNSCs (EFH) (grey bars) in control (Oct4CRE+/tk−) and experimental groups (Oct4CRE+/tk+) are assessed at day 10 (i) or day 40 (ii). The numbers of YFP+ neurospheres are indicated in yellow bars. Day 10, n = 3 mice/group for day 10; Day 40, n = 3 Oct4CRE/tk- mice, n = 5 Oct4CRE/tk + mice. All data represent mean ± SEM.

Finally, we generated triple transgenic Oct4CreER^T2^;ROSAyfp^fl/fl^;GFAPtk mice (herein called (Oct4CRE+/tk) that permit the labeling of Oct4 expressing pNSCs with TAM induction, as well as the selective ablation of dNSCs using AraC + GCV, to examine the *in vivo* repopulation of dNSCs. Neonatal mice received TAM labeling from PND1-14 followed by a 4 week chase. Mice sacrificed at 6 weeks of age, prior to AraC + GCV treatment, had 4.2–7.4% of pNSC-derived colonies expressing YFP+ in both control (Oct4CRE+/tk−) and experimental (Oct4CRE+/tk+) groups. Young adult mice received AraC + GCV treatment and were sacrificed immediately following ablation or 30 days later (day 10 and day 40, respectively). YFP+, pNSC-derived colonies were seen in control (9% on day 10; 16% on day 30) and experimental mice (14.89% on day 10; 15.31% on day 40) (Fig. 4bi–iii). As predicted, dNSCs were completely ablated in the CRE+/tk+ group (Fig. 4ci). Most interesting, YFP + dNSC-derived neurospheres were seen at day 40 in CRE+/tk+ mice revealing their derivation from Oct4 + pNSCs labeled prior to ablation (Fig. 4cii). These *in vivo* labeling studies reveal that Oct4 + pNSCs can give rise to GFAP + dNSCs.

**Figure 5:**
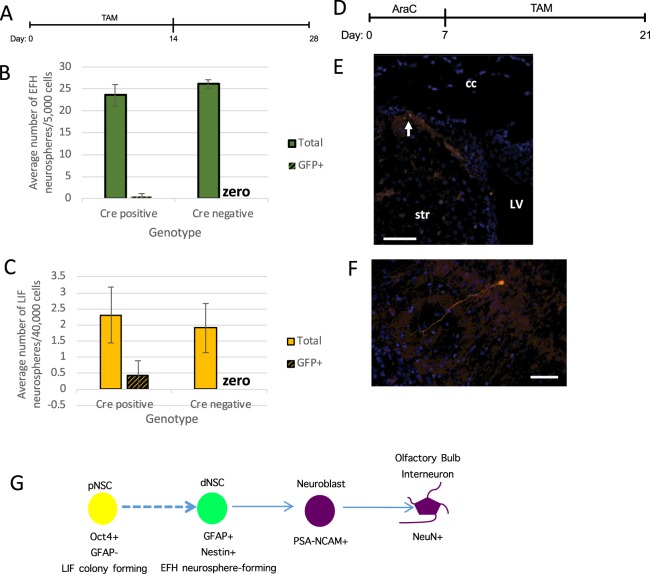
Oct4 expressing, primitive NSC contribution to the neural lineage. (**a**) Schematic of the experimental paradigm. (**b**) Neurosphere assay performed from Oct4CreERT2;tdTomatofl mice that had been fed tamoxifen assayed for dNSCs derived from pNSCs (Cre positive) against controls (Cre negative). (**c**) The number of pNSC derived colonies labeled assessed in Oct4CreERT2;tdTomatofl mice (Cre positive) versus controls (Cre negative). (**d**) Schematic of the experimental paradigm. (**e**) Rare tdTomato+, Oct4 expressing cells are located in the dorsolateral corner of the lateral ventricles during repopulation following ablation with AraC of the dNSCs and progeny. Scalebar = 100 µm. LV = lateral ventricle, str = striatum, cc = corpus callosum. (**f**) tdTomato+ neurons located in the olfactory bulb derived from Oct4 expressing pNSCs. Scalebar = 100 µm. (**g**) Proposed lineage where dNSC in the adult brain SVZ are GFAP expressing cells that form clonally derived neurospheres in the presence of growth factors (EGF and FGF and heparin)(EFH). These dNSC proliferate to give rise to migrating neuroblasts on the rostral migratory stream. Neuroblasts migrate to the olfactory bulb where they differentiate into olfactory bulb interneurons. Rare GFAP negative, Oct4 positive, LIF responsive, colony-forming cell are found in the periventricular region of the adult brain. These pNSCs are found during development, as early as E5.5, and persist into adulthood.

To further confirm the lineage relationship between pNSCs and their downstream progeny, we used Oct4CreERT2;tdTomatofl mice and subjected them to the 7 days AraC ablation paradigm (Suppl. Fig. 1). In a first series of experiments, Oct4CreERT2;tdTomatofl mice were fed tamoxifen through their mother’s milk for PND0-14 and allowed to chase until young adults (Fig. 5a). Neurosphere assays performed following this recombination paradigm demonstrated that only a rare population of pNSCs are labeled in these mice using tamoxifen-induced recombination (Fig. 5b,c). In Oct4CreERT2;tdTomatofl mice that received ablation, despite the rarity of pNSCs in adulthood, following tamoxifen food (Fig. 5d) we found tdTomato+ cells in the subependyma (Fig. 5e). Most interesting, we also observed mature neurons in the olfactory bulb expressing tdTomato, indicating they were derived from pNSCs (Fig. 5f). pNSCs may give rise to more differentiated progeny directly without going through a dNSC state in addition to giving rise to dNSCs (Fig. 5g). Excitingly, this confirms that pNSCs give rise to all cells in the neural lineage and reside at the top of the neural stem cell hierarchy.

## Discussion

Lineage tracing has demonstrated the *in vivo* relationship between the two NSC populations found in the mouse forebrain. Using several transgenic mouse models that permit the selective labeling of dNSCs and pNSCs in the forebrain subependyma, and the selective ablation of dNSCs, we demonstrate that pNSCs are upstream of the dNSC population. This lineage relationship was demonstrated in naïve mice as well as after dNSC ablation.

Under baseline conditions, we have shown that pNSCs generate cells of all three lineages during embryogenesis, and that the progeny are widespread throughout the brain parenchyma. Interestingly, we found that the majority of pNSC progeny are neuronal in the early post-natal brain. This suggests that pNSCs from E16-18 are more likely to expand symmetrically and generate neuronal progeny than give rise to dNSCs or glial progenitors during this time. The fact that we observed precursors in the periventricular region of the PND21 brain suggests that the pNSCs generated progeny that became quiescent *in vivo*. Importantly, the dNSCs that arise from pNSCs are maintained into adulthood. Understanding the direct connection between pNSCs and dNSCs and their capacity to generate all neural cell types is key in positing predictions about their behavior post-ablation and their potential to contribute to neural repair post-injury. Recent findings have identified pNSCs along the entire neuraxis of the developing and adult mouse CNS, and further, spinal cord injury models have demonstrated that pNSCs can migrate to the site of injury in the injured spinal cord^13^. This raises the possibility that pNSCs can respond to migratory cues and could contribute to neural repair.

The lack of increase in the size of the pNSC pool suggests that pNSCs divide asymmetrically to generate dNSCs during repopulation. While we have shown that pNSCs can give rise to dNSCs, it remains possible that pNSCs give rise directly to neuronal progeny without going through a dNSC state. Indeed, the fact that pNSCs transplanted onto the rostral migratory stream give rise to neuroblasts and neurons in the olfactory bulb supports this lineage relationship^7^. Further, we cannot rule out the possibility that a more quiescent subpopulation of dNSCs also contributes to the subependymal repopulation post-ablation^14^, and this would be consistent with the observation that differential levels of GFAP protein expression in dNSCs has been demonstrated *in vivo*^15^. Notably, while differential GFAP expression could explain why no YFP expressing dNSC-derived neurospheres were seen following ablation (i.e. if the GFAP expression was too low to be depleted by the GCV), it cannot explain the current observation that Oct4+ (YFP expressing) cells give rise to dNSCs *in vivo*. Hence, while our work demonstrates that pNSCs give rise to dNSCS, we cannot rule out the possibility that pNSCs can bypass the dNSC state and give rise directly to lineage-committed cells during development or in the adult during SVZ repopulation.

Recent work has demonstrated that pNSCs and dNSCs are differentially regulated populations of neural stem cells that exist along the entire neuraxis; in both neurogenic (forebrain) and aneurogenic regions (spinal cord)^9,13^. Further it has been suggested that the dNSC population present in the adult is derived from a developmental population and set aside early in development^16^, which is consistent with the idea that pNSCs are most abundant during development^7^ and present as early as E7.5^6^. Additionally, another group has shown that the gene expression of the dNSC population exists on a continuum throughout development and adulthood^17^, which falls in line with our data demonstrating pNSCs persisting into adulthood. We propose that pNSCs give rise to dNSCs during development and persist as a reserve source of stem cells in the mature CNS, which can be activated in response to injury^4,7^.

## Methods

### Mice

Mice were housed within the Department of Comparative Medicine at the University of Toronto. The University Animal Care Committee (UACC) and Research Oversight and Compliance Office at the University of Toronto approved the research ethics, in accordance with the guidelines of the Canadian Council of Animal Care prior to experimentation. The following transgenic mice, both sexes, were used in this study. GFAP-TK Line 7.1, a kind gift from Dr. M Sofroniew (UCLA), bred in house. GFAP-CreER^T2^ and GFAP-gfp, a kind gift from Dr. A Nagy (University of Toronto), bred in house. Rosa26-EYFP^fl^ (http://jaxmice.jax.org/strain/006148.html), Pou5f1^tm1.1(Cre/Esr1)Yseg^ (https://www.jax.org/strain/016829) and B6;129S6-Gt(ROSA)26Sor^tm9(CAG-tdTomato)Hze^/J (https://www.jax.org/strain/007905) purchased from The Jackson Laboratories (USA). Combinations of the strains are crossed to generate 1) GFAPCreER^T2^;ROSAyfp^fl/fl^; GFAPtk, 2) ROSAyfp^fl/fl^; GFAPtk, 3) Oct4CreER^T2^;ROSAyfp^fl/fl^;GFAPtk and 4) Oct4CreER^T2^;ROSA26-tdtomato^fl/fl^ mice. Pregnant CD-1IGS mice (http://www.criver.com/products-services/basic-research/find-a-model/cd-1-mouse) (Charles River (Canada)) were used for fostering.

### Dissection and *in vitro* assays

The neurosphere assay was performed as previously described^18^. Briefly, mice were killed with an overdose of avertin or isoflurane, and the brain was removed. The periventricular region lining the lateral ventricle was dissected and dissociated into single cells using enzymatic treatment (trypsin (1.33 mg/ml) and hyaluronidase (0.67 mg/ml) and kynurenic acid (0.2 mg/ml)- Sigma-Aldrich, St. Louis, MO) and mechanical trituration. Cells are plated at clonal density (10 cells/ul) (a density at which spheres are derived from single starting cells^19^) in serum free media containing l-glutamine (2 mM, Invitrogen), penicillin/streptavidin (100 U/0.1 mg/ml, Invitrogen), mitogens (epidermal growth factor (EGF, 20 ng/ml); fibroblast growth factor (FGF, 10 ng/ml); heparin (2 ug/ml), Sigma) for dNSCs; or leukemia inhibitory factor (LIF, 10 ng/ml) for pNSCs). EFH is used to denote the dNSC media conditions. Primary neurospheres were counted after 7 days with the following strict inclusion criteria: pNSCs > 50 µm and dNSCs > 80 µm in diameter.

### Tamoxifen labelling

To label the Oct4+ cells during development timed pregnant female Oct4CreER^T2^;ROSA26-Tdtomato^fl/fl^ were injected with tamoxifen (Sigma) on embryonic day 16 and once daily for 3 days (E16-18). Tamoxifen was prepared as previously described^20^ in a suspension of 10% ethanol in 90% sunflower oil at a concentration of 30 mg/ml. Each mouse received an intraperitoneal injection of 3 mg/day taking care to avoid the developing fetuses. Fetuses were delivered by cesarian section, fostered to CD1 lactating dams and allowed to survive for 3 weeks.

For labeling cells in adult animals, young 4 week-old GFAPCreER^T2^;ROSAyfp^fl/fl^;GFAPtk mice were fed tamoxifen chow (250 mg/kg, Envigo) for 2 weeks followed by a 2-week chase. For Oct4CreER^T2^;ROSAyfp^fl/fl^ and Oct4CreER^T2^;ROSA26-Tdtomato^fl/fl^ mice, post-natal day (PND)1 pups are fostered to CD-1 mothers that had age matched litters. CD-1 mothers were fed tamoxifen chow (250 mg/kg, Envigo) for 2 weeks, followed by a 4 week chase.

### Ablation paradigms

Mice were anaesthetized with 5% isoflurane (inhalation) and ketoprofen (5 mg/kg; injected SQ). Alzet 1007D mini osmotic pumps (0.05 ul/hour, Direct Corp.) containing 2% L-arabinose cytarabine (AraC) were implanted subcutaneously and attached to a cannula implanted into the lateral ventricle at the coordinates: 0.2 mm AP from bregma, 0.7 mm ML, and 2.5 mm DV below the dura. AraC was delivered for 7 days followed immediately by sacrifice, tamoxifen chow for two weeks, or infusion of ganciclovir (GCV) (200uM, Sigma) for 3 days.

### Injection cannulation and retrovirus injection

ROSAyfp^fl/fl^;GFAPtk mice were anaesthetized with 5% isoflurane (inhalation) and ketoprofen (5 mg/kg; injected SQ). Injector cannula (C315G2-2-SP Guide, Plastics One) was placed in the lateral ventricle of the opposite hemisphere to the mini osmotic pumps and secured to the surface of the skull with dental cement. Retrovirus carrying Cre-Recombinase (in-house^21^) was injected through the cannula daily at 3uL for 3 days during GCV infusion.

### Tissue preparation and immunohistochemistry

Wholemount sections derived from Oct4CreER^T2^;ROSA26-tdtomato^fl/fl^ mice as previously described^7,22^. Briefly, animals were overdosed as described and perfused with PBS. Brains were removed and the walls of the lateral ventricles were exposed. Wholemounts were fixed for 48 hours in 4% PFA followed by 48 hours in 1x PBS for washing prior to incubation with Hoescht (Sigma B2261, 1:1000) in PBS for 24 hours. Wholemounts of the entire lateral wall of the ventricle were removed and mounted on superfrost glass slides, coverslipped and allowed to settle for 48 hours before visualization.

Mice that received tamoxifen injections in utero were perfused on PND21 as previously described^7^. Briefly, animals were overdosed with tri-bromo-ethanol (Avertin, Sigma) and transcardially perfused with ice cold PBS followed by 4% paraformaldehyde. Brains were removed and post-fixed for 4 hours in 4% paraformaldehyde and subsequently transferred to 20% sucrose in PBS for cryoprotection. Brains were frozen and serially sectioned on a cryostat to derive 30 µm sections on superfrost slides (Fisherbrand, Fisher Scientific). Sections were stained with Hoescht (1:1000, Sigma) in PBS for 20 minutes to visualize tdtomato+ cells. For immunohistochemistry, slides were rehydrated in PBS for 10 min followed by a 1 hour incubation at room temperature with 10% NGS in 0.3% Tritonx100 in PBS. Sections were incubated overnight at 4 °C with primary antibodies in 10% NGS in PBS. Primary antibodies were GFAP (Sigma; rabbit polyclonal, 1:500; or DAKO, rabbit polyclonal, 1:2000), Olig2 (Millipore, rabbit polyclonal, 1:500), SOX2 (Abcam, rabbit polyclonal, 1:2000), NeuN (Abcam, rabbit polyclonal, 1:2000), and MBP (Abcam, rat monoclonal, 1:50). Following washing, sections were incubated with secondary antibodies Alexa Fluor 488 goat anti-rabbit IgG or goat anti-rat IgG with Hoescht (1:1000, Sigma) in 10% NGS in PBS for one hour at room temperature. Sections were washed and mounted using Mowiol (in house) and left to dry at room temperature overnight. Visualization of tissue was performed on a Zeiss spinning disc confocal using Zen software for acquisition or an inverted confocal microscope with a motorized stage (Olympus FV1000). The percentage of tdTomato expressing cells that co-labeled with GFAP, Olig2, Sox2, or NeuN at PND21 was calculated from sections evenly distributed throughout the brain (n = 4 mice). In addition, the location of tdTomato+ cells was recorded (n = 4 mice).

### Statistics

Data are represented as mean ± SEM. Two-tailed t-tests were performed to compare between two groups. ANOVA was used to compare between multiple groups with Dunnet’s post hoc test. Significance was considered p < 0.05. All graphs and analysis were generated from either Excel (Microsoft) or GraphPad Prism 6 (Graph Pad Software, California).

## Supplementary information

Supplementary Information
