# Supplementary material for: Lineage tracing reveals the hierarchical relationship between neural stem cell populations in the mouse forebrain
Source: Sci Rep. 2019 Nov 27;9:17730. doi: 10.1038/s41598-019-54143-9 (PMC6881290; doi:10.1038/s41598-019-54143-9)
Supplement: Supplementary file 1 — Supplementary Information [file 41598_2019_54143_MOESM1_ESM.pdf]

Lineage tracing reveals the hierarchical relationship between neural stem cell populations in the mouse forebrain

Nadia Sachewsky[1*], Wenjun Xu[1,2*], Tobias Fuehrmann[4], Derek van der Kooy [2,3] and Cindi M Morshead[1,2,4,5]

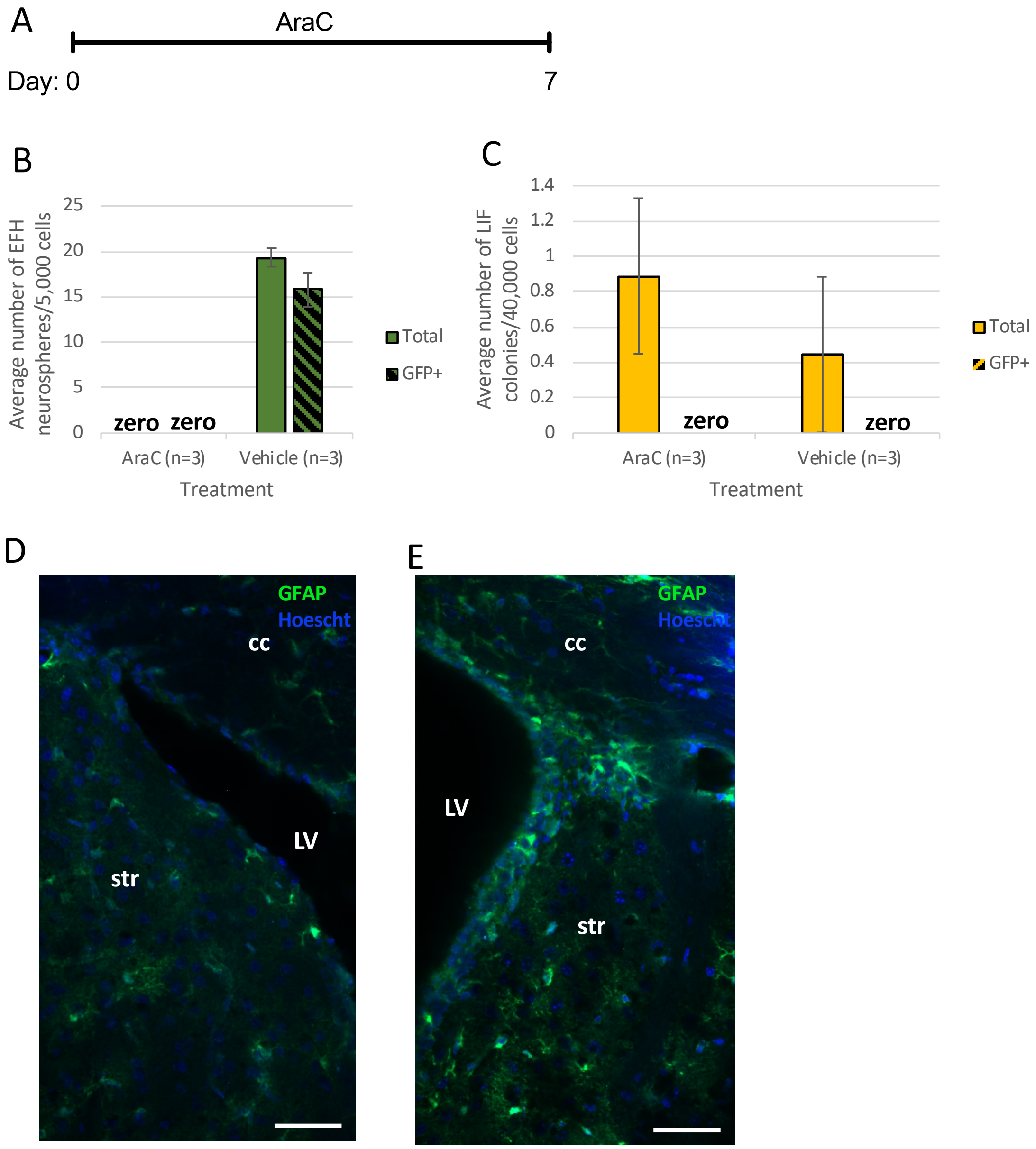
A
AraC
Day: 0
7
B
Average number of EFH neurospheres/5,000 cells
25
20
15
10
5
0
zero zero
AraC (n=3)
Vehicle (n=3)
Treatment
Total
GFP+
C
Average number of LIF colonies/40,000 cells
1.4
1.2
1
0.8
0.6
0.4
0.2
0
zero
zero
AraC (n=3)
Vehicle (n=3)
Treatment
Total
GFP+
D
GFAP
Hoescht
cc
LV
str
E
GFAP
cc
LV
str

Supplemental Figure 1: GFAP expressing dNSCs are ablated following AraC infusion

a, Schematic of the experimental paradigm**.** **b,** Number of dNSC derived neurospheres (EFH) following ablation with AraC or vehicle infusion in GFAP-gfp mice (n=3 per group) and the number that are gfp+. **c**, pNSC derived colonies (LIF) derived following ablation with AraC or vehicle infusion in GFAP-gfp mice (n=3 per group) and the number that are gfp+. **d,** Lateral ventricle image from sections of ablated mice with loss of GFAP expressing cells in the subependyma of GFAP-gfp mice. **e,** Lateral ventricle image from sections of vehicle infused mice with normal distribution of GFAP expressing cells in the subependyma of GFAP-gfp mice. Scalebar = 100μm. LV = lateral ventricle, str = striatum, cc = corpus callosum. Green = GFAP, Blue = Hoescht.
